# PAR-4/Ca^2+^-calpain pathway activation stimulates platelet-derived microparticles in hyperglycemic type 2 diabetes

**DOI:** 10.1186/s12933-021-01267-w

**Published:** 2021-04-03

**Authors:** Alessandra Giannella, Giulio Ceolotto, Claudia Maria Radu, Arianna Cattelan, Elisabetta Iori, Andrea Benetti, Fabrizio Fabris, Paolo Simioni, Angelo Avogaro, Saula Vigili de Kreutzenberg

**Affiliations:** Metabolic Disease Unit, Department of Medicine–DIMED, Via Giustiniani, 2, 35128 Padova, Italy

**Keywords:** Platelet activation, Extracellular vesicles, NF-*k*B, Glycated hemoglobin, THP-1 transformed macrophages

## Abstract

**Background:**

Patients with type 2 diabetes (T2DM) have a prothrombotic state that needs to be fully clarified; microparticles (MPs) have emerged as mediators and markers of this condition. Thus, we investigate, in vivo, in T2DM either with good (HbA1c ≤ 7.0%; GGC) or poor (HbA1c > 7.0%; PGC) glycemic control, the circulating levels of MPs, and in vitro, the molecular pathways involved in the release of MPs from platelets (PMP) and tested their pro-inflammatory effects on THP-1 transformed macrophages.

**Methods:**

In 59 T2DM, and 23 control subjects with normal glucose tolerance (NGT), circulating levels of CD62E+, CD62P+, CD142+, CD45+ MPs were determined by flow cytometry, while plasma levels of ICAM-1, VCAM-1, IL-6 by ELISA. In vitro, PMP release and activation of isolated platelets from GGC and PGC were investigated, along with their effect on IL-6 secretion in THP-1 transformed macrophages.

**Results:**

We found that MPs CD62P^+^ (PMP) and CD142^+^ (tissue factor-bearing MP) were significantly higher in PGC T2DM than GGC T2DM and NGT. Among MPs, PMP were also correlated with HbA1c and IL-6. In vitro, we showed that acute thrombin exposure stimulated a significantly higher PMP release in PGC T2DM than GGC T2DM through a more robust activation of PAR-4 receptor than PAR-1 receptor. Treatment with PAR-4 agonist induced an increased release of PMP in PGC with a Ca^2+^-calpain dependent mechanism since this effect was blunted by calpain inhibitor. Finally, the uptake of PMP derived from PAR-4 treated PGC platelets into THP-1 transformed macrophages promoted a marked increase of IL-6 release compared to PMP derived from GGC through the activation of the NF-*k*B pathway.

**Conclusions:**

These results identify PAR-4 as a mediator of platelet activation, microparticle release, and inflammation, in poorly controlled T2DM.

**Supplementary Information:**

The online version contains supplementary material available at 10.1186/s12933-021-01267-w.

## Background

Hyperglycemia promotes systemic inflammation and platelet dysfunction in patients with type 2 diabetes (T2DM) [1]. Glycated hemoglobin (HbA1c), an indicator of metabolic control, is an independent predictor of cardiovascular complications [2], and associates positively with atherosclerosis, ischemic heart disease, stroke, and hypertension [3]. Platelet function is markedly altered in T2DM but is reversed by HbA1c correction [4]. Moreover, HbA1c levels correlate with the expression of P-selectin [5], a soluble marker of platelet activation, and also with an enhanced number of reticulated platelets, indicating an accelerated thrombopoiesis in T2DM [6].

Hyperglycemia is also a potent stimulator of microparticles (MPs) formation [7–9]. The MPs are a heterogeneous population of membrane vesicles of 100–1000 nm diameter, generated by various stimuli including hyperglycemia, apoptosis, proinflammatory cytokines, oxidative stress, infectious agents from several cell types, of which MPs maintain the surface and cytoplasmic markers [10]. MPs deliver bioactive molecules, like microRNA or inflammation mediators, from cell to cell, thus mediating the exchange of biological information and regulating pathophysiological responses and signaling pathways [10, 11].

In type 2 diabetes mellitus, several microparticle types have been described, even in the early phase of the disease [8, 9, 12], and in the pre-diabetic condition [13]. Circulating MPs are linked with poor metabolic control and micro- and macrovascular complications [9, 14–16]. MPs released by activated or apoptotic platelets, via plasma membrane surface budding, represent the prevalent population of circulating MPs and are significantly increased in diabetes [17]. Platelet-derived MPs (PMP) actively participate in the inflammatory and atherosclerotic process; once internalized by monocytes or endothelial cells, they upregulate cytokines and intercellular adhesive molecular-1 expression [18], favor leukocyte migration [19], and reduce nitric oxide (NO) levels [20]. The release of specific miRNAs from PMPs has been suggested as potential non-invasive biomarkers of platelet function in T2DM [21]. Yet, the mechanisms underlying the propensity of hyperglycemia to induce MP release by platelets are mainly unknown.

With this background in mind, we aimed to determine the effects of chronic hyperglycemia on platelet-derived MPs formation in humans (primary end-point), and additionally, to translationally investigate the mechanisms involved in PMP release, along with their potential contribution to chronic inflammation.

## Methods

### In vivo study

#### Study subjects

We recruited 59 consecutive T2DM, 43 men and 16 women attending the outpatient clinic of the Division of Metabolic Diseases of the University of Padova, from March to November 2017. Inclusion criteria were: type 2 diabetes diagnosis according to the ADA criteria; both genders; age 18–80 years. Exclusion criteria were: type 1 diabetes, clinically relevant diseases, or advanced chronic diabetes complications. According to the values of glycated hemoglobin (HbA1c), T2DM were divided into 2 groups, i.e. with good (mean HbA1c ≤ 7.0%; GGC; n = 28) and poor (mean HbA1c > 7.0%; PGC; n = 31) glycemic control. A control group of matched subjects with normal glucose tolerance (NGT) ascertained by an oral glucose (75 g) tolerance test was included in this study.

The primary demographic and anthropometric data, duration of diabetes, blood pressure values, heart rate, and current therapy were recorded in all the subjects.

A fasting blood sample was drawn by venipuncture from an antecubital vein, in each patient for the determination of glucose, HbA1c (only in T2DM), lipid profile (total cholesterol, high-density lipoprotein cholesterol, low-density lipoprotein cholesterol, triglycerides), hematocrit, hemoglobin, red and white blood cell count, platelet count, IL-6, ICAM-1, VCAM-1, and for the assessment and characterization of circulating MPs. Platelets were also collected from T2DM for the in vitro studies.

This study was carried out under the International Ethical Guidelines and the principles of the Declaration of Helsinki and was approved by the local Institutional Review Board of the University of Padova Medical Centre. All subjects signed informed consent.

#### Biochemical analyses

Plasma glucose, total serum cholesterol, triglyceride, and HDL cholesterol were measured using standard enzymatic methods. LDL cholesterol was calculated by the Friedewald formula. HbA1c was measured via high-performance liquid chromatography. Chromatography was performed using a certified automated HPLC analyzer; the normal range was from 4.25 to 5.9% (23 to 41 mmol/mol).

Red blood cells, hematocrit, hemoglobin, white blood cells, and platelet count were determined by standard methods. According to the manufacturer’s instructions, plasma levels of IL-6, VCAM-1, and ICAM-1 were measured by using a high-sensitivity ELISA assay (BioVision, CA, USA). The intra and inter-assay coefficients of variations were below 10%. All samples were coded for a blinded analysis, and each plasma sample was determined in duplicate.

#### Circulating microparticles assessment and characterization

Activated endothelial cells MPs (CD62E^+^), tissue factor-bearing MPs (CD142^+^), leukocyte-derived MPs (CD45^+^), and activated platelet-derived MPs (CD62P^+^, PMP) were determined. Microparticles were prepared from platelet-free plasma (PFP) within 3 h of blood collection by double centrifugation (3000×*g* for 15 min). One ml of PFP was centrifuged at 18000×*g* for 40 min at 4 °C to obtain microparticles. MPs were resuspended in 200 µL of phosphate-buffered saline (cat# D8537, PBS, Sigma, USA) and stored at − 80 °C until use. Samples, analyzed only after a single freeze–thaw cycle, were thawed by incubation for 5 min in a water bath at 37 °C immediately before assay.

All assays were performed on a Cytomics FC500 flow cytometer (Beckman Coulter, Miami Florida), as previously described [13]. The MPs gate was established using a blend of mono-dispersed fluorescent beads of three diameters (0.5, 0.9, and 3 μm) (cat# 7801, Megamix, BioCytex, Diagnostica Stago, France) [22]. Twenty microliters (µL) of freshly thawed MPs were directly incubated for 15 min at room temperature in the dark with 2 µL of fluorescent-conjugated monoclonal antibodies against cell-type specific antigens and 2 µL of annexinV-FITC (fluorescein isothiocyanate) (cat# BMS500FI, Bender MedSystems GmbH, Vienna, Austria) for 20 min at 37 °C. Endothelial-derived MPs were identified using CD62E-PE (phycoerythrin) (cat# 336008, BioLegend Europe, The Netherlands) and platelet-derived MPs using CD62P-PC5 (phycoerythrin-cyanin 5.1) (cat# 304908, BioLegend Europe, The Netherlands); leukocyte-derived MPs using CD45-PC5 (cat# 304009, BioLegend Europe, The Netherlands) and Tissue Factor-bearing (TF^+^ MPs) with CD142-PE, clone HTF-1 (cat# 550312, BD, Biosciences, Milan, Italy). The isotype controls used were IgG1-PC5, clone MOPC-21 (cat# 400118, BioLegend Europe), IgG1-PE, clone MOPC-21 (cat# 556650, BD Biosciences, Milan, Italy); mouse IgG1-FITC, clone MOPC-21 (cat# 400129, BioLegend Europe). The samples were diluted in 500 μL of 0.22 µm filtered Annexin-V kit binding buffer (Bender MedSystems GmbH, Vienna, Austria) before analysis. A total of 20 μL of counting beads with an established concentration (cat# 7547053, Flow Count™ Fluorospheres, Beckman Coulter, Miami Florida) were added to each sample to calculate MPs as absolute numbers per microliter. Patient samples were all processed in the same way by the same experienced operators.

### In vitro study

#### Platelet preparation and platelet-derived microparticle (PMP) production

Platelets were isolated from fresh human blood by centrifugation at 200×g for 20 min at 20 °C to obtain platelet-rich plasma (PRP). Washed platelets were isolated from PRP after centrifugation and resuspended in calcium and magnesium-free HEP buffer with prostaglandin E1 (1 µM, pH 7.4). Platelet counts were determined with a cell counter (TC20™, Biorad, USA). In vitro, platelet-derived microparticles were generated from platelets (500 × 10^3^ platelets/µL), incubated for 30 min at 37 °C with thrombin (1 U/mL), or with PAR-1 (20 µM), or PAR-4 (200 µM) agonists, and collagen (10 μg/mL) co-stimulus, or with calcium ionophore A23187 (10 μmol/L) in Tyrode’s buffer (1 mM MgCl_2_, 2 mM CaCl_2_, 3 mM KCl), as previously described [23]. Activation was stopped by the addition of 2.0 mmol/L of EDTA. Platelets and debris were removed with centrifugation for 10 min at 3000×*g* and PMP were obtained after centrifugation and analyzed by flow cytometer, as described above.

#### *Platelet cytosolic Ca*^*2*+^*measurement*

Ca^2+^ measurement was determined as previously described [24]. Briefly, platelets (1.5 × 10^7^ cells/μL) were loaded with the fluorescent probe, 2.5 μM Fura-2/AM, for 30 min at 37 °C. After recovery, levels of cytosolic calcium (Ca_cyt_^2+^) were measured by Shimadzu spectrofluorometer. The baseline fluorescence was obtained by alternating the excitation wavelength between 340 and 380 nm and recording the 510 nm emission intensity. [Ca^2+^]_I_ was calculated from the fluorescence ratio recordings according to the standard formula: [Ca^2+^]_I_ = Kd [(R − R_min_)/(R_max_ − R)](Sf2/Sb2). The dissociation constant (Kd) was taken as 224 nmol/L; R_max_ (340/380 ratio under Ca^2+^-saturating conditions), R_min_ (340/380 ratio under Ca^2+^-free conditions;), and Sf2/Sb2 (ratio of baseline fluorescence) were calculated by a calibration curve with buffers containing different Ca^2+^ concentrations; PAR-1 agonist (20 µM) or PAR-4 agonist (200 µM) were added when baseline fluorescence was stable. Basal Ca_cyt_^2+^ levels were reported after a 60 s recording period. All determinations were performed in duplicate for each patient.

#### Western Blotting

Platelets were lysed in RIPA buffer containing protease inhibitors. Proteins were separated by 10% SDS-PAGE and electrophoretically transferred onto a nitrocellulose membrane in a semidry blotter. Blots were incubated for 1 h with Tris-buffered saline containing 0.1% Tween 20 and 5% skimmed milk to block residual protein binding sites. Membranes were incubated overnight with specific antibodies against anti-PAR-1 (1:1000; cat# ab233741, Abcam, Cambridge, UK), anti-PAR-4 (1:1000; cat# 2328S, Cell Signaling Tech. MA, USA), and anti-β actin (1:5000; cat# 3700, Cell Signaling Tech. MA, USA). Detection was achieved using an enhanced chemiluminescence system (cat# EMP011005, Euroclone, Italy). The blots were scanned and quantified using a chemiluminescence molecular imaging system (Versa Doc 3000. Bio-Rad, Hercules, CA, USA). The results were expressed relative to the control on the same blot, defined as 100%, and by the protein of interest/β actin densitometric ratio. Protein concentration was determined by BCA’s method (cat# EMP014500, Euroclone, Italy).

#### Calpain activity assay

Calpain activity was determined by Calpain-Glo™ Protease Assay (cat# G8502, Promega, Madison, USA) according to the manufacturer’s instructions. Briefly, washed platelet (400 to 500 × 10^9^ platelets/L) were stimulated with PAR-4 agonist peptide (AY-NH_2_) for 30 min at 37 °C in the presence and in the absence of ALLN, a calpain inhibitor. Then, all conditions were centrifuged (10 min at 650×*g*) and the pellets were resuspended in lysis buffer (20 mM Tris–HCl, pH 7.5, 1 mM EDTA, 1 mM dithiothreitol, and Protease Inhibitor Cocktail) for 30 min at 4 °C. Lysates (50 μL) were mixed with 50 μL of Calpain-Glo™ Reagent (with 2 mM CaCl_2_ for calpain activation) incubated for 30 min at room temperature and finally the luminescent readings performed in an EnSight™ multimode plate reader (Perkin Elmer, Milan, Italy). All the results were expressed as relative luminescence units per microgram of protein lysate.

#### PMP staining and incorporation into macrophages

The THP-1 cell line was obtained from American Type Culture Collection (Manassas, VA, USA). Cells were cultured in RPMI-1640 medium (Sigma Aldrich, Milan, Italy) supplemented with 10% FBS, 1% l-glutamine, and 1% antibiotic solution in a humid atmosphere containing 5% CO_2_ at 37 °C. For the induction of macrophage differentiation, cells (1–2 × 10^6^ per mL) were seeded with 100 nM phorbol 12-myristate 13-acetate (PMA, cat# 79346, Sigma Aldrich, Milan, Italy) for 72 h. After incubation, nonattached cells were removed by aspiration, and the adherent cells were washed and cultured in serum-reduced RPMI-1640 medium (3% FBS). Subsequently, THP-1 transformed macrophages were treated with PMP (1000 MPs/µL). To assess the uptake of PMP in THP-1, PMP were stained with 10 µL of Calcein-AM for 40 min (20 µM; cat#17783, Sigma Aldrich. Milan, Italy), washed and resuspended in PBS for the incorporation into macrophages (0.5 × 10^6^ cells), as previously described. Calcein-AM is non-fluorescent until enters into intact MPs to be activated and becomes fluorescent (λ_ex_ 496 nm; λ_em_ 516 nm ± 5 nm). After 4 h, cells were washed once with PBS, fixed with paraformaldehyde 4% and counterstained with 4,6-diamidino-2-phenylindole (DAPI) (cat# D9542, Sigma Aldrich. Milan, Italy; λ_ex_ 340 nm; λ_em_ 488 nm). Images were captured using a Zeiss microscope (Oberkochen, Germany) with Apotome upgrade for confocal imaging (630×).

Moreover, THP-1 cells were incubated with no labelled PMP (1000 MPs/µL) generated from PAR-4 treated platelets of T2DM with PGC (in the presence and in the absence of ALLN, a calpain inhibitor) and with GGC. Treatment with unstimulated PGC-PMP and TNFα (10 ng/mL) on THP-1 were performed as a negative and positive control, respectively. After 24 h, THP-1 cells and their culture medium were collected to measure the gene expression and release of IL-6, and NF-kB acetylation [25].

#### Cell viability assay

At the end of each treatment, the number of live and total cells was counted with trypan blue staining (Sigma Aldrich. Milan, Italy). Cell viability was assessed by calculating the percentage of live cells using trypan blue exclusion.

#### Gene expression

##### RNA extraction

Total RNA was isolated from THP-1 transformed macrophages by RNeasy Mini kit (cat# 74104, Qiagen, Hilden, Germany), following the manufacturer’s instructions. RNA was treated with DNase I (Roche) before reverse transcription (RT). RNA was quantified using the NanoDrop 2000C (Thermo Scientific, USA). cDNA was synthesized with 500 ng of RNA extracted using iScript cDNA synthesis kit (cat#1708891, Bio-Rad, Hercules, CA) according to the manufacturer’s instructions. Quantitative real-time polymerase chain reaction assay was performed in a Bio-Rad CFX96 Real-Time PCR detection system. The PCR reaction was performed in a 25 µL final reaction volume containing 200 nmol of each primer and SsoFast EVAGreen SuperMix (cat# 5201, Bio-Rad, USA). All the reactions were performed in 96-well plates, in triplicate. Primers were designed from sequences derived from the GenBank database using Primer 3 (Whitehead Institute, Massachusetts, USA) and Operon's Oligo software (Operon, California, USA). They were purchased from Eurofins MWG (Ebersberg, Germany). The specific primers were (Eurofins): IL-6, Forward AGTCCTGATCCAGTTCCTGC and reverse CTACATTTGCCGAAGAGCCC; β-actin, as a housekeeping gene, Forward AGAGCTACGAGCTGCCTGAC and reverse GGATGCCACAGGACTCCA. Data analyses were performed with the Bio-Rad CFX Manager. The comparative cycle threshold method (∆∆Cq) was used to obtain the relative fold change of gene expression.

#### IL-6 quantification

Interleukine-6 (IL-6) levels were quantified in cell culture supernatants, using a commercially available enzyme-linked immunosorbent assay (Raybio Human IL-6 ELISA kit; RayBiotech Norcross, GA, USA), following the manufacturer's protocols.

#### NF-kB acetylation

NF-*k*B-acetylation was carried out by incubating 2 μg of anti-NF-*k*B antibody with 1 mg of cell lysate overnight, followed by 30 μg of EZ viewTM Red Protein A Affinity Gel (cat# P6486, Sigma Aldrich. Milan, Italy) for 4 h at 4 °C. After washing, immunoprecipitates were boiled in SDS-PAGE loading buffer, subjected to SDS-PAGE, transferred on to nitrocellulose filters and probed with the specified primary antibody against acetylated lysine (cat# 9814, Cell Signaling Tech. MA, USA) and the appropriate horseradish peroxidase-conjugated secondary antibody (GE Healthcare, Illinois, USA). Results were expressed relative to the control, on the same blot, and the values were expressed as fold increase after normalization with total NF-*k*B.

#### PAR-1 and PAR-4 agonists

PAR-1 agonist peptide (TRAP-6, [serine-phenylalanine-leucine-leucine-arginine-asparagine amide], cat# 3497) and PAR-4 agonist peptide (AY-NH_2_, H_2_ [alanine–tyrosine–proline–glycine–lysine–phenylalanine amide], cat# 1487) were purchased from Tocris. Calpain Inhibitor I (ALLN, cat# A6185) was purchased by Sigma Aldrich.

### Power analysis

We used previous data [8], to calculate the sample size needed in order to estimate the statistically significant difference between PMP in subjects with type 2 diabetes and non-diabetic subjects. Since there are no data about differences in patients either in good or poor metabolic control, we assumed no difference between normal subjects and T2DM in good metabolic control. Considering an α error level of 5%, 16 subjects per group will allow for an estimate of the difference between groups with a power equal to 90%.

### Statistical analysis

Continuous variables are expressed as mean ± SEM and categorical variables as percentages. Data were tested for significance using a Student’s t-test for two normally distributed groups. Variable normality distribution was performed by the Shapiro–Wilk test. Data from three or more groups were analyzed by one-way ANOVA test followed by a Bonferroni post hoc test. Categorical data were analyzed with a Chi-squared test. To determine the association between MPs type and studied variables, univariate analyses were run. Statistical significance was accepted at p < 0.05. SPSS (IBM SPSS Statistics for Windows, version 26 Bologna, Italy) and GraphPad (vers. 0.8.3 for Mac, La Jolla, CA) were used for statistical analysis.

## Results

### In vivo study

The study subject main demographic and anthropometric parameters, blood pressure, biochemical determinations and ongoing therapies are reported in Table 1. According to the values of glycated hemoglobin (HbA1c), T2DM were divided into two groups, i.e. with good (mean HbA1c ≤ 7.0%; GGC) and poor (mean HbA1c > 7.0%; PGC) glycemic control. This cut-off value was decided considering current guidelines recommendation for preventing or delaying micro- and macrovascular complications. PGC patients had a higher platelet count, increased IL-6, and VCAM-1 levels, than GGC patients and NGT. There were no differences in the two groups regarding all other studied parameters. Control subjects showed significantly lower BMI, glucose level, systolic blood pressure, IL-6, ICAM-1, and VCAM-1 compared to T2DM (Table 1).

**Table 1:** Main clinical characteristics of the study subjects

Parameters	NGT (n 23)	T2DM (n 59)	p value	GGC T2DM (n 28)	PGC T2DM (n 31)	p value
Sex (M/F)	15/8	43/16	0.54	20/8	23/8	0.28
Age (years)	58 ± 2	60 ± 1	0.33	60 ± 2	58 ± 2	0.09
Diabetes duration (years)	–	11.3 ± 0.9	–	10.1 ± 1.3	12.6 ± 1.2	0.16
CV events (n)	–	6	–	2	4	0.67
BMI (kg/m^2^)	25 ± 0.5	29 ± 0.6	0.02	29 ± 0.8	29 ± 10.0	0.78
HbA1c (mmol/mol)	–	60.1 ± 2.0	–	47.9 ± 0.71	70.7 ± 2.5	< 0.0001
HbA1c (%)	–	7.5 ± 0.1	–	6.4 ± 0.1	8.6 ± 0.2	< 0.0001
SBP (mmHg)	122 ± 2.0	137 ± 2.0	0.04	135 ± 3.0	139 ± 3.0	0.39
DBP (mmHg)	77 ± 3.0	81 ± 1.0	0.09	83 ± 2.0	80 ± 2.0	0.17
HR (bpm)	74 ± 3.0	76 ± 3.0	0.23	76 ± 2.0	77 ± 2.0	0.61
Glucose (mg/dL)	85 ± 3.0	168 ± 7.0	0.001	154 ± 10.0	181 ± 10.0	0.06
Total cholesterol (mg/dL)	168 ± 6.0	177 ± 4.0	0.53	173 ± 6.0	181 ± 7.0	0.38
HDL cholesterol (mg/dL)	51 ± 2.0	50 ± 2.0	0.35	51 ± 2.0	49 ± 3.0	0.45
LDL cholesterol (mg/dL)	104 ± 5.0	106 ± 4.0	0.31	105 ± 5.0	108 ± 5.0	0.56
Triglyceride (mg/dL)	114 ± 10.0	122 ± 7.0	0.57	107 ± 9.0	133 ± 12.0	0.06
White blood cells (10^9^/L)	6.9 ± 0.4	6.5 ± 0.2	0.44	6.2 ± 0.2	6.7 ± 0.2	0.11
Red blood cells (10^12^/L)	4.7 ± 0.3	4.7 ± 0.1	0.76	4.6 ± 0.6	4.8 ± 0.7	0.26
Hematocrit (%)	42 ± 0.1	42 ± 0.3	0.54	43 ± 0.5	42 ± 0.5	0.41
Hemoglobin (g/L)	144 ± 2.0	143 ± 1.3	0,31	142 ± 1.8	143 ± 1.9	0.96
Platelets (10^9^/L)	210 ± 5.0	229 ± 7.0	0.07	213 ± 7.0	243 ± 12.0	0.03
IL-6 (pg/mL)	3.4 ± 0.2	4.9 ± 0.2	0.02	4.4 ± 0.2	5.4 ± 0.4	0.004
VCAM-1 (ng/mL)	658 ± 26.0	958 ± 36.0	0.01	858 ± 26.0	1032 ± 56.0	0.008
ICAM-1 (ng/mL)	173 ± 9.0	210 ± 7.0	0.03	214 ± 9.0	218 ± 12.0	0.65
Antiplatelet drugs (%)	–	47	–	52	43	0.60
ASA (%)	–	41		46	36	0.56
ADP antagonists (%)	–	7	–	4	10	0.68
Statins (%)	–	65	–	70	60	0.58
OAD, n (%)	–	68	–	78	60	0.17
Metformin (%)	–	75	–	73	76	0.81
Incretins (%)	–	26	–	23	28	0.94
Glitazone (%)	–	7	–	8	14	0.77
Sulphonylurea (%)	–	7	–	4	10	0.68
Insulin (± OAD) (%)	––	32	–	22	40	0.17
Diet	–	2	–	4	0	0.96

Data are presented as mean ± SEM. Student’s t-test or chi-squared test were applied, PGC vs. GGC)

*NGT* normal glucose tolerance, *T2DM* type 2 diabetes mellitus, *GGC* good glucose control, i.e. HbA1c ≤ 7%, *PGC* poor glucose control, i.e. HbA1c > 7%, *CV events* cardiovascular events, *BMI* Body Mass Index, *SBP* systolic blood pressure, *DBP* diastolic blood pressure, *HR* heart rate, *bpm* beats per minute, *HDL* high density lipoprotein, *LDL* low density lipoprotein, *IL-6* Interleukin 6, *VCAM-1* Vascular Cell Adhesion Molecules-1, *ICAM-1* Intercellular Adhesion Molecules-1, *ASA* acetylsalicylic acid, *OAD* oral antidiabetic drugs

**Fig. 1:**
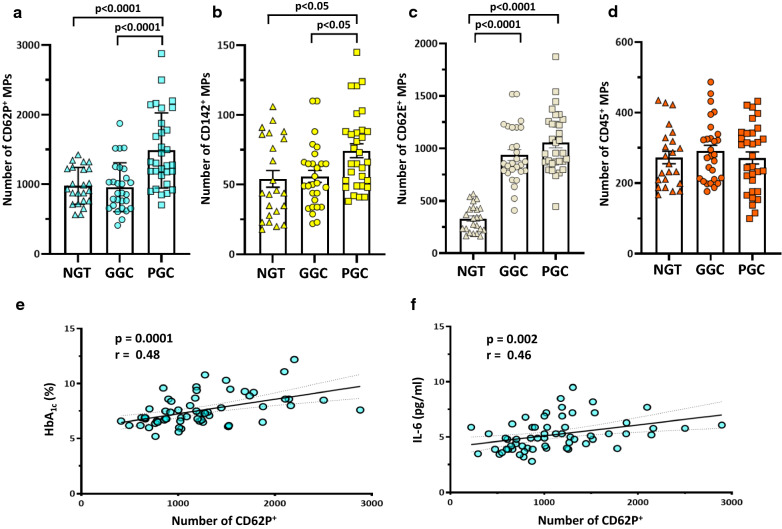
Circulating microparticles characterization in plasma from T2DM. Plasma-derived microparticles were isolated from 23 subjects with normal glucose tolerance (NGT), 28 diabetic patients with HbA1c ≤ 7.0% (GGC) and 31 diabetic patients with HbA1c > 7.0% (PGC). **a**–**d** Microparticles were labelled with specific antibodies: **a** for platelet-derived MPs (CD62P^+^) (ANOVA, p < 0.0001), **b** for tissue factor bearing (CD142^+^) (ANOVA, p = 0.009), **c** for endothelial-derived MPs (CD62E^+^) (ANOVA, p < 0.0001), and **d** for leukocyte-derived MPs (CD45^+^) (ANOVA, p = 0.64). Values are mean ± SEM. The p-values were evaluated by ANOVA followed by a *post-hoc* Bonferroni test. **e**, **f** Correlations between CD62P^+^ MPs and glycated hemoglobin, and between CD62P + MPs and IL-6 plasma levels, respectively. Statistical significance was determined with linear regression. Dotted lines indicated the 95% of interval confidence

First, we determined the circulating MPs: activated platelet-derived MPs (CD62P^+^; PMP), tissue factor-bearing MPs (CD142^+^; TF-MPs), activated endothelial cells MPs (CD62E^+^), leukocyte-derived MPs (CD45^+^) (Fig. 1a–d). We found that PMP and TF-MPs were significantly increased in plasma from PGC T2DM, compared to GGC or to NGT subjects (Fig. 1a, b). CD62E^+^ MPs were significantly increased in T2DM compared to NGT but were not affected by glucose control (Fig. 1c). On the other hand, no difference was observed in CD45^+^ MPs levels across different groups (Fig. 1d). Moreover, only PMP showed a positive correlation with both HbA1c and IL-6 (Fig. 1e, f), while TF-MPs showed a correlation with HbA1c values (r = 0.33; p = 0.02), but not with IL-6 plasma levels (r = 0.151; p = 0.25). We did not observe any significant correlation between PMP and fasting glucose level in all the groups (Additional file 1: Fig. S1).

We also evaluated the effect of antiplatelet therapy on MPs release, and found no effect between T2DM with or without antiplatelet drugs on PMP (1208 ± 112 vs. 1256 ± 91 respectively; p = 0.737), on endothelium-derived MPs (1029 ± 60 vs. 944 ± 47; p = 0.268), and on leukocyte-derived MPs (284 ± 17 vs. 286 ± 24; p = 0.961). Only TF-MPs were significantly reduced in patients taking an antiplatelet drug (56.4 ± 5.7 vs. 76.5 ± 6.5; p = 0.026). Considering these results, we then focused our research on PMP.

### In vitro study

#### Effect of thrombin and PARs agonists on the release of PMP from human platelets according to Hb1Ac

**Fig. 2:**
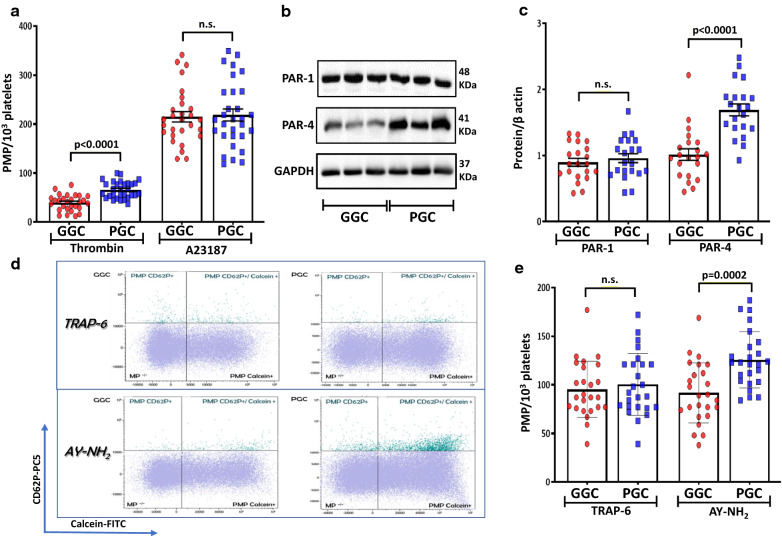
PAR-4 receptor expression increased the release of PMP from diabetic patients with PGC. **a** Platelets from T2DM with GGC (HbA1c ≤ 7.0%, n = 28) and with PGC (HbA1c > 7.0%, n = 30) were incubated for 30 min with thrombin (1 U/mL) and collagen (10 µg/mL) co-stimulus, or with Ca^2+^ ionophore A23187 (10 µM). PMP were isolated and were analyzed by flow cytometer. **b**, **c** Representative Western blots and densitometric analysis of PAR-1 and PAR-4 protein expression in platelets from T2DM with GGC and with PGC. The results were expressed relative to the control on the same blot, defined as 100%, and by the protein of interest/β actin densitometric ratio (n = 21). **d** Representative scatter plot of MPs derived from platelets of (left*)* GGC and (right) PGC T2DM treated with (up*)* TRAP-6 (PAR-1 agonist, 20 μM) and (down) AY-NH_2_ (PAR-4 agonist, 200 μM), labelled with CD62P (PMP), and Calcein-AM; double positive MPs are shown (PMP CD62P^+^/Calcein^+^); CD62P^+^ MPs (PMP) are shown in green, CD62P^−^ MPs are shown in light violet. **e** Numbers of PMP released by platelets from T2DM with GGC and with PGC (n = 25), treated with TRAP-6 and with AY-NH_2_, determined by flow cytometry. Values are mean ± SEM; The p-values were evaluated by t-test

In vitro, PMP release can be induced in platelets by thrombin or by A23187, a calcium ionophore; thrombin binds the protease-activated receptor (PAR) family while A23187 acts in a receptor-independent manner. We, therefore, determined the effects of thrombin and A23187 on PMP release, detected as CD62P^+^ by flow cytometry, from platelets of TD2M with good (HbA1c ≤ 7.0%; GGC) and poor (HbA1c > 7.0%; PGC) glucose control. Figure 2a shows that thrombin induced a higher release of MPs from platelets of PGC compared to GGC T2DM; on the other hand, this effect was not observed with A23187 treatment. These results suggest a receptor-dependent effect of thrombin on the release of PMP, and that platelets from PGC T2DM are more susceptible and more promptly to generate MPs in comparison with platelets from GGC T2DM.

Protease-activated receptors (PARs) play a key role for platelet activation mediated by thrombin [26, 27]. Human platelets contain large amounts of PAR-1 and PAR-4 receptors [28]. Therefore, we tested the hypothesis that the increased release of circulating PMP in T2DM according to the glucose control involves the PARs expression. First, we measured the protein expression of PAR-1 and PAR-4 in platelets from GGC and PGC T2DM. PAR-4 protein expression was significantly increased in platelets from PGC compared to GGC T2DM, while PAR-1 protein expression did not differ significantly between the two groups (Fig. 2b, c). Furthermore, we verified that the effect on PARs is mainly due to a chronic state of unbalanced glucose metabolism rather than to acute hyperglycemia, since the expression of PAR-1 and PAR-4 in platelets did not change in a subgroup of healthy controls in comparison to GGC (Additional file 1: Fig. S2a–c). Then, to provide experimental evidence that PARs can differentially influence the release of MPs from platelets, we measured the effects of TRAP-6, a PAR-1 agonist, and of AY-NH_2_, a PAR-4 agonist, on the production of PMP from platelets of GGC and PGC T2DM. As we expected, AY-NH_2_ treatment induced a higher PMP release from platelets of PGC in comparison to GGC T2DM. On the other hand, the effect of TRAP-6 treatment on PMP release was similar in the platelets from the two groups (Fig. 2d, e). Finally, we did not observe significant differences in the release of PMP in platelets treated with AY-NH_2,_ between and GGC and NGT groups (Additional file 1: Fig. S2d).

#### Role of calpain in the release of MPs in platelets

**Fig. 3:**
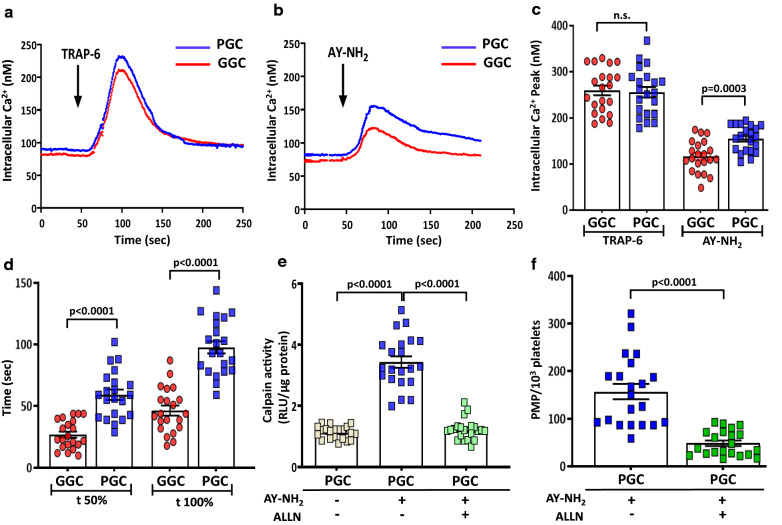
Effect of PAR agonists on Ca^2+^ mobilization and calpain activity in platelets from T2DM. **a**–**d** Effect of PAR agonists on intracellular Ca^2+^ in platelets of GGC and PGC T2DM. Cells were loaded with Fura-2 and placed in a thermostated cuvette in a physiological buffer. **a**, **b** Representative tracing of intracellular Ca^2+^ (Ca^2+^_i_) from platelets, by fluorimetric measurements. TRAP-6 (PAR-1 agonist, 20 μM) and AY-NH_2_ (PAR-4 agonist, 200 μM) were added when baseline fluorescence was stable. Red lines indicate Ca^2+^_i_ of platelets of GGC T2DM, and blue lines indicate Ca^2+^_i_ of platelets of PGC T2DM. **c**, **d** Ca^2+^ peak values, and time of Ca^2+^ recovery in platelets from T2DM with GGC and with PGC, respectively. t 50%, half-time of recovery; t 100% total time of recovery. **e** Calpain activity in platelets from T2DM with PGC, stimulated with AY-NH_2_ (200 μM) and in the presence of calpain inhibitor (ALLN, 100 μM). Calpain activity was determined as the value of luminescence recorded as relative light units (RLU) per µg of protein lysate. **f** Counts of platelets-derived microparticles (PMP) released by platelets from T2DM with PGC treated with AY-NH_2_, (PAR-4 agonist), in the presence and in absence of calpain inhibitor, ALLN (n = 21). Values are mean ± SEM. The p-values were evaluated by t-test (**c**, **d**, **f**) or ANOVA (p < 0.0001) followed by a *post-hoc* Bonferroni test (**e**)

Since PARs belong to the family of G-protein-coupled receptors, and their binding with thrombin induces Ca^2+^ mobilization, we verified whether the increase of PAR-4 mediated PMP release from PGC T2DM platelets may be due to a Ca^2+^-dependent mechanism. To test this hypothesis, we measured the effects of PAR-4 and PAR-1 agonists on Ca^2+^ mobilization in platelets from T2DM with different levels of Hb1Ac. Figure 3a, b show representative fluorimetric traces of intracellular Ca^2+^ (Ca^2+^_i_) induced by PAR-1 and PAR-4 agonists in platelets from GGC and PGC T2DM. The stimulation with PAR-1 agonist generated a very rapid initial peak of Ca^2+^ mobilization, which then quickly returned to basal value (Fig. 3a). However, no difference in the calcium response was seen between the two groups after PAR-1 agonist stimulation (Fig. 3a, c). On the contrary, Ca^2+^ mobilization evoked by PAR-4 agonist showed a slower onset and a sustained signal trend (Fig. 3b), describing a higher peak of Ca^2+^ mobilization (Fig. 3c) with an increase of the half time and of the complete Ca^2+^ recovery in platelets of PGC than GGC (t50%, 58 ± 3 vs. 29 ± 2 s; t100%, 96 ± 6 vs. 49 ± 3 s; Fig. 3d). Basal Ca^2+^ was similar in platelets of the two groups (GGC 85 ± 8 nM; PGC 91 ± 6 nM; p = 0.12).

Next, we explored possible mechanisms by which Ca^2+^ mobilization induced by PAR-4 is responsible for the increased PMP release in T2DM with PGC. Since PMP are released by cytoskeletal anchoring and require calpain, a calcium-regulated cysteine proteinase, we determined calpain activity in platelets stimulated by PAR-4 agonist (AY-NH_2_). Since the activity of calpain is significantly increased after PAR-4 stimulation in platelets from PGC compared to GGC and NGT (Additional file 1: Fig. S2d), we performed a simultaneous treatment with ALLN, a calpain inhibitor in platelets from PGC. We showed a strong inhibitory effect of ALLN on calpain activity induced by PAR-4 agonist (Fig. 3e).

Then, to support the hypothesis that calpain also affects PMP release in PGC, we measured PMP derived from platelets stimulated with PAR-4 in the presence and absence of ALLN. As shown in Fig. 3f, the marked increase of PMP induced by PAR-4 agonist was entirely abolished by ALLN, underlying the calpain pivotal role in releasing PMP in T2DM with poor glycemic control.

#### Role of PMP on the secretion of IL-6

To explore the biological consequences of PMP released by PAR-4 treated platelets from GGC or PGC T2DM, we determined: first, the ability of THP-1 transformed cells to incorporate these MPs and, second, the possibility that incorporated PMP may regulate the activation of intracellular pro-inflammatory pathways [29].

**Fig. 4:**
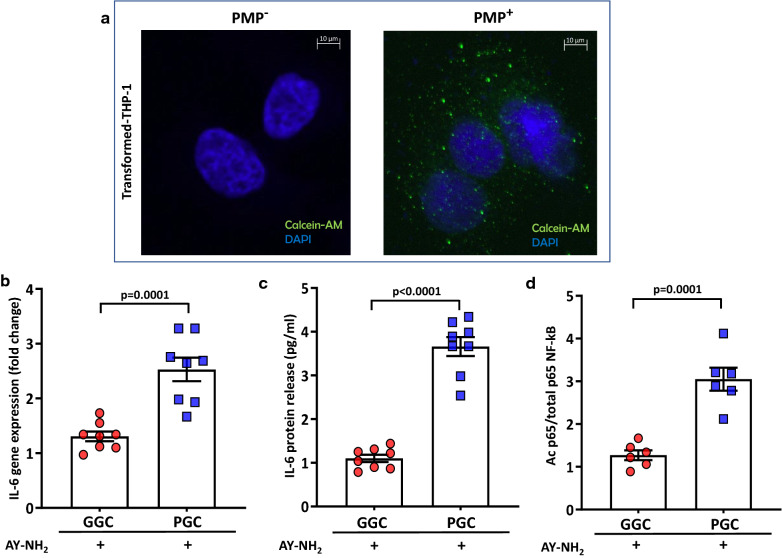
Effect of PMP from PAR-4 treated platelets on IL-6 release and NF-*k*B activation in THP-1. Uptake of microparticles (1000 MPs/µL) into THP-1 transformed macrophages obtained from PAR-4 treated platelets of T2DM with GGC and with PGC for 24 h. **a** Representative confocal fluorescence images obtained from Zeiss microscopy implemented with the ApoTome attachment (×630) of PMP into THP-1 transformed macrophages, labelled with Calcein-AM (1 µM) for 40 min and co-cultured with macrophages for 4 h. Images of THP-1 (left) in absence and (right) in presence of PMP treatment. Calcein-AM^+^ MPs are shown in green, while the nucleus is stained in *blue* with DAPI. **b**–**d** THP-1 cells were incubated for 24 h with PMP from platelets of GGC and PGC T2DM, treated with AY-NH_2_ a PAR-4 agonist. **b** IL-6 gene expression was measured by qPCR, and **c** IL-6 release was measured by ELISA assay (n = 8). **d** NF-*k*B activation was determined as the acetylation level of p65 subunit (Ac p65 NF-*k*B) normalized for p65 NF-*k*B protein (total p65 NF-*k*B) (n = 6). Values are mean ± SEM. The p-values were evaluated by t-test

**Fig. 5:**
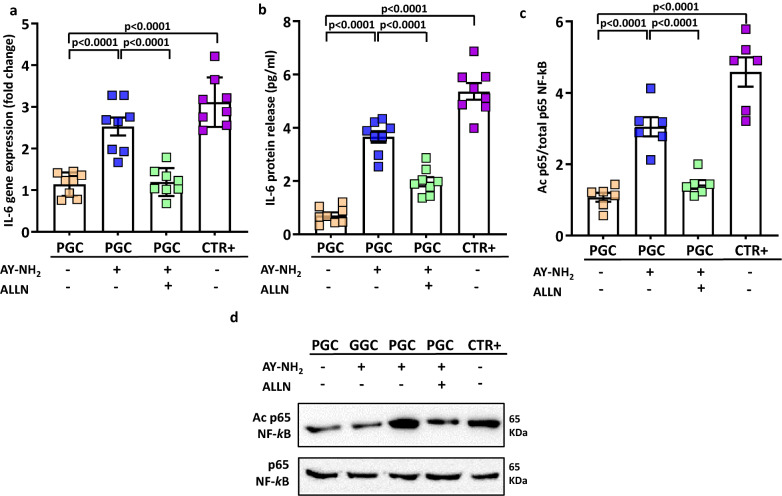
Effect of PAR-4 treated-platelet MPs on IL-6 and NF-*k*B in THP-1, blunted by calpain inhibition. THP-1 cells were incubated for 24 h with PMP from PGC T2DM, unstimulated and treated with AY-NH_2_ a PAR-4 agonist, in presence and in absence of ALLN, a calpain inhibitor. **a** IL-6 gene expression was determined by qPCR, and **b** IL-6 release was measured by ELISA assay (n = 8). **c** NF-*k*B activation was determined as the acetylation level of p65 subunit (Ac p65 NF-*k*B) normalized for p65 NF-*k*B protein (total p65 NF-*k*B) (n = 6). **d** Representative Western blots of THP-1 cells incubated with PMP from platelets of GGC and PGC, treated with AY-NH2 a PAR-4 agonist; PGC platelets were also incubated in presence and in absence of ALLN. TNFα (10 ng/mL) was used as positive control. Values are mean ± SEM. The p-values were evaluated by ANOVA (p < 0.0001) followed by a post-hoc Bonferroni test

As shown in Fig. 4a, PMP labeled with Calcein-AM have been internalized into THP-1 transformed cells. The uptake of PMP from PAR-4 treated platelets into THP-1 transformed cells induced a marked increase of the gene expression of IL-6, along with an increased secretion of IL-6 when PMP are derived from PGC, but not from GGC (Fig. 4b, c). Moreover, these effects were blunted in the presence of ALLN, the calpain inhibitor (Fig. 5a, b). To gain further insight into the mechanisms by which PMP induced the secretion of IL-6 into THP-1, we investigated the activity of NF-*k*B, one of the main factors involved in the regulation of cytokines. We measured the level of acetylated NF-*k*B-p65 in THP-1 cells incubated with PMP from PAR-4-treated platelets. As shown in Fig. 4d, acetylation of NF-*k*B-p65 was significantly enhanced in THP-1 transformed cells treated with MPs from platelets of PGC T2DM, with cells treated with MPs from platelets of GGC T2DM. Furthermore, we demonstrated that in the presence of calpain inhibitor (ALLN), the effect on the acetylation of NF-kB-p65 in THP-1 transformed macrophages incubated with PMP from PGC was significantly reduced (Fig. 5c, d).

## Discussion

**Fig. 6:**
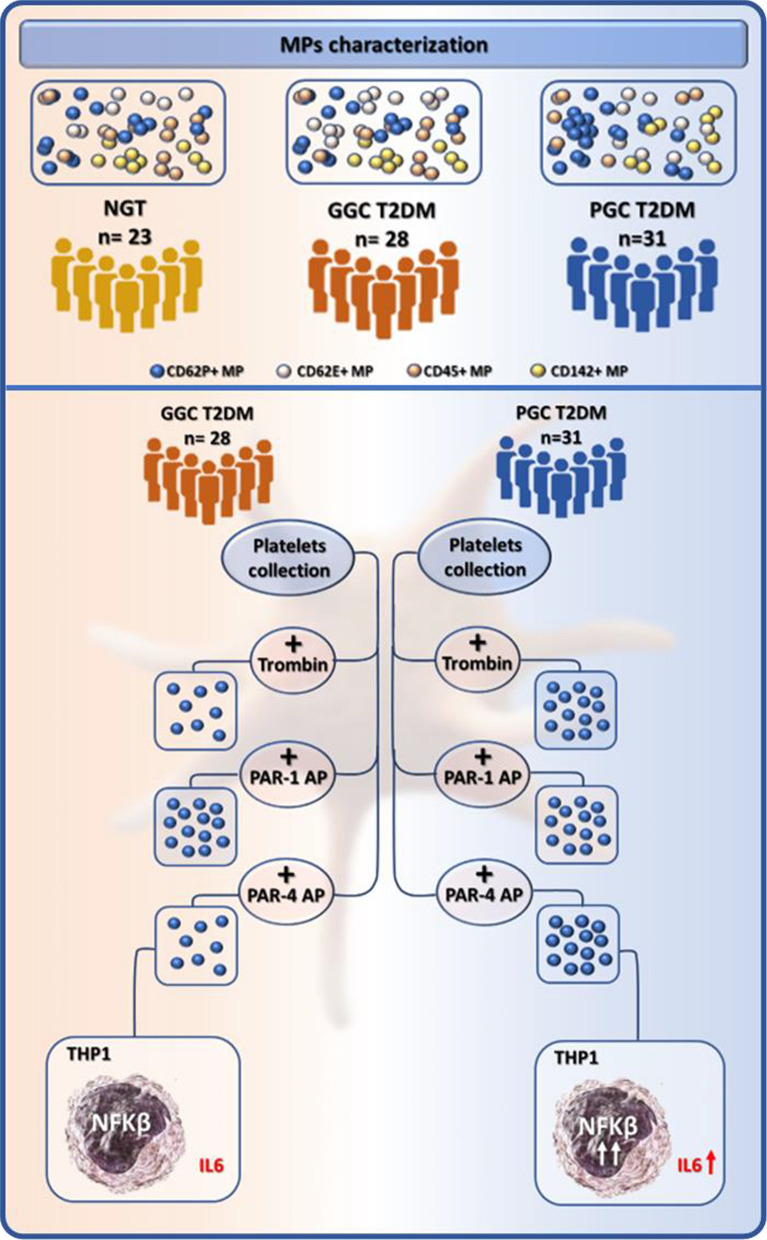
Study design and summary. In vivo, circulating Microparticles (MPs) characterization was performed in T2DM with good (HbA1c≤7.0%; GGC) or poor (HbA1c>7.0%; PGC) glycemic control. In vitro, platelets from GGC and PGC were treated with thrombin and PAR agonists (PAR1/4-AP) to generate MPs (PMP), and to investigate their molecular pathways. PMP from PAR-4 stimulated platelets were incubated into THP-1 transformed macrophages to test their pro-inflammatory effects

In this study, we found that poor metabolic control, in T2DM patients is associated with higher levels of platelet-derived MPs (CD62P^+^; PMP) and tissue factor-bearing MPs (CD142^+^; TF-MPs). We also found that circulating PMP strongly correlate with IL-6, suggesting a link between the excessive release of PMP and inflammation. For the first time, we demonstrated that, in human platelets from T2DM subjects, not only protease-activated receptor 4 (PAR-4) promotes the release of activated PMP through a Ca^2+^-calpain dependent mechanism, but also that its expression is upregulated by chronic hyperglycemia. Furthermore, in a set of in-vitro experiments in THP-1 transformed macrophages, we showed that PMP release from PAR-4 stimulated PGC platelets contributes to subclinical inflammation by stimulating IL-6 expression and secretion, via the NF-*k*B pathway (Fig. 6). These data indicate a pivotal role of PAR-4 activation on PMP release and action, in type 2 diabetes chronic hyperglycemia.

PMP represent the majority of total plasma MPs, in T2DM [30, 31], and are a marker of platelet activation, and impaired platelet function [12, 14]. Chronic hyperglycemia, possibly as a consequence of either oxidative stress-induced apoptosis [32] or the associated hypercoagulability [33, 34] enhances the release of MPs. It is well known that chronic hyperglycemia, subclinical inflammation, and enhanced platelet activation are strongly correlated to each other, and play an essential role in developing vascular complications of diabetes [35]. Our results offer further insights into the mechanisms linking platelets, MPs, and inflammation in the context of diabetes vascular complications.

We excluded possible interference of antiplatelet therapy on PMP release, in vivo, since about half of our diabetic patients were on anti-platelets treatment: while in vitro studies indicate that antiplatelet agents inhibit PMP release, clinical studies evaluating the effect of treatment with aspirin on PMP release provide inconsistent results [36]. We also observed no influence on circulating levels of PMP by antiplatelet therapy in agreement with others [37, 38], who showed that aspirin treatment did not change the level of PMP in T2DM. None of the study subjects assumed prasugrel, which shows the most potent effect on platelet inhibition in T2DM [39]. On the other hand, TF-MPs appeared to be significantly lower in antiplatelet treated patients [37].

It is interesting to note that the levels of PMP and TF-MPs were similar between T2DM with good glucose control (HbA1c < 7%) and control subjects, indicating a preeminent action of hyperglycemia on their release. At the same time, endothelium-derived MP were increased in T2DM, compared to NGT, independent of chronic glucose levels. This observation suggests that hyperglycemia is an essential stimulus for PMP and TF-MPs release, and significantly impairs platelet metabolism.

Although recent studies have suggested that antidiabetic drugs, as GLP-1 receptor agonists, and metformin could interfere with platelet activity [40, 41], the present study does not address this issue, and moreover a similar number of subjects in the two subgroups of T2DM patients assumed these therapies. A possible anti-thrombotic activity has also been suggested for SGLT-2 inhibitors [42]; however, we point out that none of our subjects was treated with these drugs.

In this study, we demonstrate that PAR-4, one of the master receptors for thrombin, but not PAR-1, is upregulated in platelets from T2DM chronically exposed to hyperglycemia. Although the specific molecular mechanisms involved in the upregulation of PAR-4 expression in T2DM are unknown, some evidence supports our findings. PAR-4 expression was increased in carotid atherectomies and saphenous vein specimens from diabetic versus nondiabetic patients, suggesting a direct role for PAR-4 in diabetic vasculopathy [43]. Moreover, Dangwal et al. demonstrated that vascular actions of thrombin, such as intracellular calcium mobilization, migration, and TNF-α gene expression are controlled through transcriptional upregulation of PAR-4, but not PAR-1, in vascular smooth muscle cell (VSMC) cultures [44]. These authors demonstrated high glucose enhances VSMC responsiveness to thrombin through upregulation of PAR-4, mediated via PKC-β, PKC-δ, and NF-*k*B. Dynamic regulation of PAR-4 expression by extracellular glucose was also described in diabetic mice and murine cardiac fibroblast cultures [45, 46]; all these studies corroborate our findings and indicate that PAR-4 expression may adapt dynamically to the stimuli such as thrombin, high glucose, and oxidative stress and can be switched on, at need, in vivo. In a mouse diabetic model, the role of PARs on platelet reactivity has been demonstrated [47]. Interestingly, PAR-4 knockout mice exhibited increased tolerance to injury, which was manifest as reduced infarct size and a more robust functional recovery compared to wild-type mice [48]. These observations suggest that platelets are critical mediators of thrombo-inflammation during reperfusion injury, and a hyperactive platelet phenotype may contribute to an exaggerated ischemia–reperfusion injury response [49].

We also observed that acute treatment with PAR-4 agonist exerted two different effects on the response of Ca^2+^ in platelets from T2DM with PGC compared to GGC: it increased Ca^2+^ peak and prolonged the time for the recovery of Ca^2+^ in PGC, suggesting the involvement of some Ca^2+^-dependent mechanisms in PMP release. The activation of the Ca^2+^-calpain pathway for the release of PMP was suggested by Pasquet et al. more than 2 decades ago [50]; in our study we tested this hypothesis and we went further, confirming the involvement of Ca^2+^-calpain pathway, in PMP release, and also showing this pathway is activated by PAR-4, and participate in the modulation of pro-inflammatory effects of PMP. In this context, it has proved that calpain inhibition attenuates atherosclerosis and inflammation through eNOS/NO/NF-*k*B pathway in an animal model [51], and markedly reduces vascular remodeling induced by Angiotensin II [52]. Calpain inhibition also suppresses IL-6 pro-inflammatory activities, in primary helper T cells and synovial fibroblasts [53], and inhibiting calpain-mediated filamin-A cleavage in macrophages impairs migration and proliferation, lipid uptake, and reduces the secretion of inflammatory interleukin-6, overall reducing atherosclerosis in mice [54].

With this background in mind, we also investigated if PMP could contribute to subclinical inflammation by evaluating their potential in stimulating IL-6 production, and whether the Ca^2+^-calpain pathway mediated this effect. Our interest was focused on IL-6, considering its master role as a pro-inflammatory and proatherogenic molecule, especially in T2DM [55], and the fact that IL-6 signaling pathway modulation by canakinumab has been demonstrated to reduce cardiovascular event rates, independent of lipid-lowering [56].

In our study, in THP-1 transformed macrophages incubated with PMP obtained from PAR-4 treated platelets of poorly controlled diabetic subjects, both the gene and protein expression of IL-6 was increased through the activation of the NF-*k*B pathway. We also observed that the Ca^2+^-calpain pathway was involved in the inflammation mediated by PAR-4 released PMP, since calpain inhibition reduced IL-6 secretion, and attenuated the release of PMP. Translating these in vitro results to our findings in vivo, we can hypothesize that PMP could contribute to the higher circulating levels of IL-6 observed in chronically hyperglycemic T2DM. To reinforce the importance of PAR-4 as a mediator of IL-6 release, recently PARs have been indicated as a possible target to treat pro-inflammatory cytokine and prothrombotic harmful effects, in COVID-19 [57], and in other pro-thrombotic conditions, as suggested in a recent Consensus Document on Atherothrombosis and Thromboembolism [58]. Prospective clinical studies are needed to verify the importance of this mechanism in the atherosclerotic process in patients with diabetes in poor metabolic control.

Study limitations. In this study, we did not measure other independent markers of in vivo platelet activation; moreover, the intra-subject reproducibility of studied parameters was not tested, by repeated blood sample collection, since the subjects were studied on one single occasion. Eventually, it would have been interesting to assess, in the PGC subjects, the effects of the restoration of glucose control: regrettably, we were unable to re-assess the patients.

## Conclusions

In conclusion, we demonstrate that PMP release and activation is enhanced by chronic hyperglycemia in humans through the upregulation of molecular pathway PAR-4/Ca^2+^-calpain, and actively contributes to IL-6 production*.* PAR-4 plays a crucial multifaceted role in the vascular complications of diabetes, and represents a possible novel target for the treatment of diabetic vascular complications.

## Supplementary Information

**Additional file 1: Figure S1.** Correlations between CD62P^+^ MPs and fasting glucose level in all groups. Statistical significance was determined with linear regression. Dotted lines indicated the 95% of interval confidence. **Figure S2.** **a**–**c** Representative Western blots and densitometric analysis of PAR-1 and PAR-4 protein expression in platelets from NGT, GGC and PGC. The results were expressed relative to the control on the same blot, defined as 100%, and by the protein of interest/β actin densitometric ratio. The p-values were evaluated by ANOVA: PAR-1, p = 0.9; PAR-4, p < 0.0001 followed by a *post-hoc* Bonferroni test. **d **Counts of platelets-derived microparticles (PMP) released by platelets from NGT, GGC and PGC treated with AY-NH_2_, (PAR-4 agonist). The p-values were evaluated by ANOVA: p = 0.006. **e** Calpain activity in platelets from NGT, GGC and PGC, stimulated with AY-NH_2_ (200 μM). Calpain activity was determined as the value of luminescence recorded as relative light units (RLU) per µg of protein lysate. The p-values were evaluated by ANOVA: p < 0.0001 followed by a post-hoc Bonferroni test. Values are mean ± SEM.

## Data Availability

The dataset(s) supporting the conclusions of this article is(are) included within the article.

## Abbreviations

ALLN: Calpain inhibitor I
AY-NH2: H2 [alanine–tyrosine–proline–glycine–lysine–phenylalanine amide]
COVID-19: Corona Virus Disease 2019
GGC: Good glycemic control
HbA1c: Hemoglobin A1c
HDL: High-density lipoprotein
ICAM-1: Intercellular Adhesion Molecule 1
IL-6: Interleukin 6
LDL: Low-density lipoprotein
MPs: Microparticles
NF-kB: Nuclear Factor-κB
NGT: Normal glucose tolerance
NO: Nitric oxide
PAR: Protease-activated receptor
PFP: Platelet-free plasma
PGC: Poor glycemic control
PMP: Platelet-derived microparticles
PRP: Platelet-rich plasma
T2DM: Patient with Type 2 Diabetes Mellitus
TF-MPs: Tissue factor-bearing MPs
THP-1: Human leukemia monocytic cell line
TRAP-6: Thrombin Receptor Activator Peptide 6
VCAM-1: Vascular Cell Adhesion Molecules-1
