# Supplementary material for: PAR-4/Ca2+-calpain pathway activation stimulates platelet-derived microparticles in hyperglycemic type 2 diabetes
Source: Cardiovasc Diabetol. 2021 Apr 3;20:77. doi: 10.1186/s12933-021-01267-w (PMC8019350; doi:10.1186/s12933-021-01267-w)
Supplement: Supplementary file 1 — Additional file 1: Figure S1. Correlations between CD62P+ MPs and fasting glucose level in all groups. Statistical significance was determined with linear regression. Dotted lines indicated the 95% of interval confidence. Figure S2. a–c Representative Western blots and densitometric analysis of PAR-1 and PAR-4 protein expression in platelets from NGT, GGC and PGC. The results were expressed relative to the control on the same blot, defined as 100%, and by the protein of interest/β actin densitometric ratio. The p-values were evaluated by ANOVA: PAR-1, p = 0.9; PAR-4, p < 0.0001 followed by a post-hoc Bonferroni test. d Counts of platelets-derived microparticles (PMP) released by platelets from NGT, GGC and PGC treated with AY-NH2, (PAR-4 agonist). The p-values were evaluated by ANOVA: p = 0.006. e Calpain activity in platelets from NGT, GGC and PGC, stimulated with AY-NH2 (200 μM). Calpain activity was determined as the value of luminescence recorded as relative light units (RLU) per µg of protein lysate. The p-values were evaluated by ANOVA: p < 0.0001 followed by a post-hoc Bonferroni test. Values are mean ± SEM. [file 12933_2021_1267_MOESM1_ESM.docx]

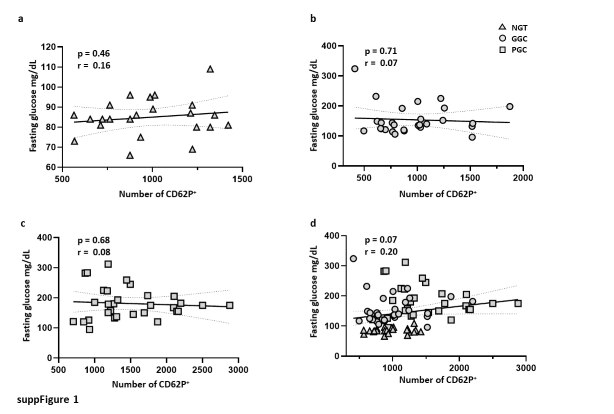
**Additional Figure S1. Correlations between CD62P^+^ MPs and fasting glucose level in all groups**. Statistical significance was determined with linear regression. Dotted lines indicated the 95% of interval confidence.


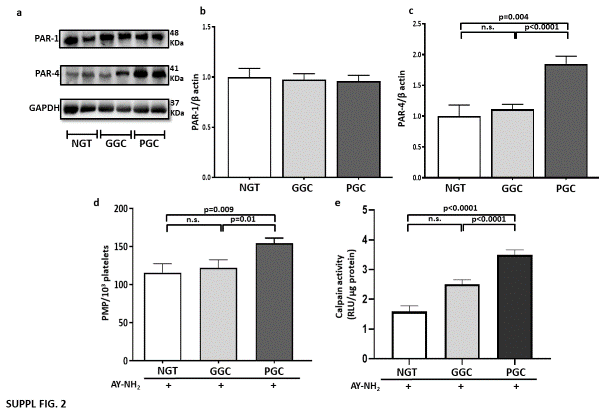


**Additional Figure S2. a - c** Representative Western blots and densitometric analysis of PAR-1 and PAR-4 protein expression in platelets from NGT, GGC and PGC. The results were expressed relative to the control on the same blot, defined as 100%, and by the protein of interest/β actin densitometric ratio. The p-values were evaluated by ANOVA: PAR-1, p = 0.9; PAR-4, p<0.0001 followed by a *post-hoc* Bonferroni test. **d** Counts of platelets-derived microparticles (PMP) released by platelets from NGT, GGC and PGC treated with AY-NH_2_, (PAR-4 agonist). The p-values were evaluated by ANOVA: p=0.006. **e** Calpain activity in platelets from NGT, GGC and PGC, stimulated with AY-NH_2_ (200 μM). Calpain activity was determined as the value of luminescence recorded as relative light units (RLU) per µg of protein lysate. The p-values were evaluated by ANOVA: p<0.0001 followed by a post-hoc Bonferroni test. Values are mean ± SEM.
